# Spatiotemporal adaptive attention graph convolution network for city-level air quality prediction

**DOI:** 10.1038/s41598-023-39286-0

**Published:** 2023-08-16

**Authors:** Hexiang Liu, Qilong Han, Hui Sun, Jingyu Sheng, Ziyu Yang

**Affiliations:** 1 College of Computer Science and Technology, Harbin Engineering University, Harbin, China; 2 Institute of Systems Engineering, Academy of Military Sciences, Beijing, 100089 China

**Keywords:** Computational models, Computational neuroscience

## Abstract

Air pollution is a leading cause of human diseases. Accurate air quality predictions are critical to human health. However, it is difficult to extract spatiotemporal features among complex spatiotemporal dependencies effectively. Most existing methods focus on constructing multiple spatial dependencies and ignore the systematic analysis of spatial dependencies. We found that besides spatial proximity stations, functional similarity stations, and temporal pattern similarity stations, the shared spatial dependencies also exist in the complete spatial dependencies. In this paper, we propose a novel deep learning model, the spatiotemporal adaptive attention graph convolution model, for city-level air quality prediction, in which the prediction of future short-term series of PM2.5 readings is preferred. Specifically, we encode multiple spatiotemporal dependencies and construct complete spatiotemporal interactions between stations using station-level attention. Among them, we design a Bi-level sharing strategy to extract shared inter-station relationship features between certain stations efficiently. Then we extract multiple spatiotemporal features with multiple decoders, which it is extracted from the complete spatial dependencies between stations. Finally, we fuse multiple spatiotemporal features with a gating mechanism for multi-step predictions. Our model achieves state-of-the-art experimental results in several real-world datasets.

## Introduction

Air pollution is a harmful substance mixed in the air in various gaseous (i.e., O_3_ and SO_2_) and particulate matter (i.e., PM). With an estimated nearly 12% of global disease deaths in 2019 being directly or indirectly caused by air pollution^1^, the negative impact of air pollution on public health and the environment has made it increasingly become the focus of science^1,2^. Policymakers monitor pollution concentrations in real-time by establishing air monitoring stations to keep abreast of regional pollution. In addition, air quality prediction is vital to reducing human disease. Accurate air quality prediction can assist policymakers in scientifically regulating corporate pollutant emissions, thereby reducing the concentration of pollutants in the air.

Differently from long-term predictions, which focus on capturing long-term dependencies in the temporal domain, we prefer to focus on accurate short-term air quality predictions. However, it is difficult to extract spatiotemporal features effectively, due to the complex spatiotemporal dependencies among air quality monitoring stations. Many researchers have worked on accurate air quality prediction and have made significant progress. Earlier studies focus on the temporal evolution of individual stations^3–7^ using traditional methods or Long Short-Term Memory neural networks (LSTM) to model the temporal trends of linear, or nonlinear relationships between sequences. The prediction accuracy of these methods is limited as they do not consider the interactions between stations. Some efforts^8–10^ have been made to leverage potential correlations between different stations, which reveal the importance of spatial correlation for air quality prediction. Due to the high construction costs of air quality monitoring stations, the number of monitoring stations is relatively small and scattered in various locations in the city. Some recent studies^11–13^ attempt to use graph convolutional networks (GCN) to model the non-Euclidean between stations. In the methods based on GCN, all monitoring stations of the whole city are regarded as nodes in the graph, and correlations correspond to the graph's edges. Multiple specific types of inter-station relationships are used to model complex spatiotemporal dependencies in air quality prediction. Each specific inter-station relationship represents a spatial dependence, such as spatial proximity, functional similarity, or temporal pattern similarity.

**Figure 1:**
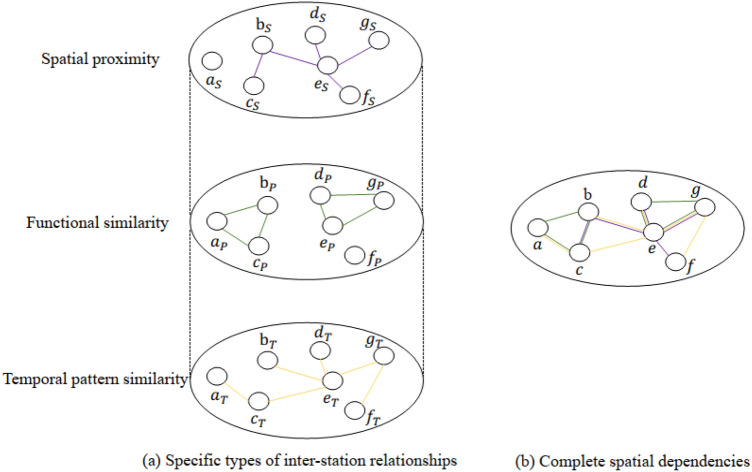
Diagram of spatial dependency. (**a**) shows three specific inter-station relationships, that is, spatial proximity (using purple to denote sparse connectivity relationship), functional similarity (green), and temporal pattern similarity (yellow). The different inter-station relationships are independent of each other. There is lacking the overall consideration of inter-station relationships. (**b**) shows the complete spatial interaction relationship in the real world, as shown in the figure, where stations may be subject to the common effects of specific inter-station relationships.

Although modeling multiple specific types of inter-station relationships contributes to the prediction accuracy, there are combinations of shared inter-station in the complete spatial dependencies. For stations, Fig. 1a,b, respectively, show the three specific types of inter-station relationships and the complete spatial dependencies. According to observations, combinations of shared inter-station are found between certain stations. For instance, stations b and c, which are geographically close with strong spatial proximity dependency, also have functional similarity dependence due to the influence of urban functional area planning. So, there are shared roles between stations b and c. Due to the diversity of combinations of shared inter-station, seven types of inter-station relationships in total may exist in reality, including spatial proximity, functional similarity, temporal pattern similarity, spatial adjacency-functional similarity, spatial adjacency-temporal pattern similarity, functional similarity-temporal pattern similarity, and spatial adjacency-functional similarity-temporal pattern similarity. Combining multiple inter-station relationships would be meaningless for extracting the above spatial features due to missing complete inter-station relationship interactions, making the extraction challenging.

To address the above challenges, we propose a novel deep learning model, the spatiotemporal adaptive attention graph convolution network model (STAA-GCN), for city-level air quality prediction. STAA-GCN captures multiple spatiotemporal features efficiently from the complex spatiotemporal dependencies and uses the encoder-decoder architecture to generate multi-step predictions. First, seven parallel encoder sets were used to encode all inter-station relationships for the complete spatiotemporal dependency construction. We design spatiotemporal relationship extraction units for specific and shared relationships, respectively. Considering complete station interaction can be correlated with either seven inter-station relationships or their combinations. We use a station-level attention mechanism to learn the interaction process. In addition, we consider the dynamic, diffusible nature of air pollution, which can lead to unequal dynamic dependencies between stations. For this reason, we design multi-angle soft attention to adaptively capture the dynamic influence relationships between stations from multiple perspectives. For multi-step predictions, Multiple inter-station relationships are decoded from the complete spatiotemporal dependencies between stations and fused using a gating mechanism at each time step. The main contributions of this paper are as follows:

- We propose a novel deep learning model. We constructed complete spatiotemporal dependency using seven parallel encoder sets and station-level attention mechanisms, from which we effectively extracted multiple spatiotemporal features.
- We design a multi-angle soft attention mechanism, which captures unequal dependencies between stations from multiple perspectives.
- STAA-GCN automatically learned shared inter-station relationships between certain stations via a Bi-level sharing strategy.
- We test our model on three publicly available datasets, and the experimental results show the superiority of our model.

## Related work

### Air quality prediction

Air quality forecasting is receiving increasing attention due to the deteriorating air environment. Existing works can be broadly classified into three categories, namely classical physical methods^14,15^, traditional methods^3,4^, and deep learning methods^5,7^. The classical physical models are based on the principle of atmospheric dispersion and use many relevant factor data to evolve the pollutant dispersion process. It is well known that there are complex correlations between relevant factors, which make it difficult to establish their numerical functions and lead to suboptimal prediction results. The traditional models, often with multi-source heterogeneous data, are employed to model temporal trends of linear between sequences. However, these methods are built from the temporal evolution of individual stations, in which spatial dependencies between stations are ignored.

Several recent studies have made significant progress that used deep learning methods to model spatiotemporal dependence^6,8,16,17^. Extensive use of feedforward neural networks for fusing influential features from other relevant stations. In addition^10,18^, consider the correlation of inequality between stations and calculate the weights of the hidden states of stations within a certain geospatial distance using an advanced attention mechanism. Some researchers^11–13^ have treated the spatial dependence between stations as non-Euclidean and used them to model the correlation between air quality stations. Although existing models such as ATGCN^11^ describe multiple spatial dependencies by constructing multi-graph, it is suboptimal due to the missing analysis of shared space dependencies.

### Multi-graph for spatiotemporal prediction

Multi-graph based methods have been widely used in spatiotemporal prediction for constructing multiple spatial dependencies^19,20^. The core of the multi-graph approach is to simultaneously learn multiple spatial dependencies by constructing multiple graphs. Existing multi-graph methods can be roughly divided into two categories: generate more graph structures to capture more detailed spatial dependencies and integrate multiple relational features more effectively.

In the first category, there are multiple correlations between stations, and pair-wise correlations are encoded as multiple graphs to capture more detailed spatial relationships. ST-MGCN^21^ believes that besides spatially adjacent, spatial dependencies also include functionality similar and transportation connected, multiple corresponding graph structures are constructed to describe a variety of spatial relationships. ATGCN^11^ coded the relationships among air quality stations as spatial adjacency, functional similarity, and temporal pattern similarity into multiple graphs, a parallel codec architecture is used for multi-step prediction.

In the second category, STAG-GCN^22^ explores Multi-layer stacked information fusion method in graph convolution, where dynamic graph features are used to automatically fuse information from each layer of static graph. DMGA-GNN^13^ first uses Spatial Attention to capture the contextual correlation of nodes in different graphs and then uses Graph Attention to obtain autocorrelation of nodes in different graphs. Finally, the gating mechanism is used to consider further the above two effects of node correlation in different graphs.

Table1 shows the comparative characteristics of existing methods and STAA-GCN. Different from other multi-graph methods, STAA-GCN relies on the construction of complete spatiotemporal dependency, which form specific and shared types of spatiotemporal relationships are extracted, rather than extracting dependencies by generating a graph for each dependency.

**Table 1:** The comparative characteristics of STAA-GCN and existing methods.

Methods	Spatial dependencies categories	Construction of spatial relationship	Aggregate multiple spatial relationships
ST-MGCN^21^ A multi-graph based network for Ride-hailing Demand prediction, multiple convolutions are used to extract spatial features and then fuse them at each time step	3	Each relation corresponds to a graph structure	Sum the spatial relationships
ATGCN^11^ A network that uses multiple sets of encoders and decoders to build and extract spatiotemporal features	3	Each relation corresponds to a graph structure	Attention mechanisms integrate spatial features
STAG-GCN^22^ The method of using dynamic graph features to fuse the feature information of each layer in the static graph is used for traffic flow prediction	3	Each relation corresponds to a graph structure	Adaptively fuse multi-layer spatial features of static graphs using dynamic graph features
DMGA-GNN^13^ A new dynamic multi-graph fusion method for spatiotemporal prediction	5	Each relation corresponds to a graph structure	Spatial Attention, graph Attention and gating mechanism
STAA-GCN The method proposed in this paper	7	In addition to graph, there is also extraction of shared relationships	Attention mechanisms to construct complete spatiotemporal and then extract the spatiotemporal relationships

## Problem formulation

We consider the multi-source heterogeneous data used in previous research^6,11,18^. Similarly, we use air quality, weather, points of interest (POI), and temporal information data for the city-wide station air quality prediction. In this section, we first describe the multi-source heterogeneous data and then formally represent the prediction.

### Monitoring data

Suppose there are $n$ air quality monitoring stations within the city, and we use the set $S=\{{s}_{i}{\}}_{i=1}^{n}$ to denote all monitoring stations. For each monitoring station, ${s}_{i}$ collects reading data for multiple pollutants (e.g., PM2.5, PM10, O_3_, NO_2_, SO_2_) and multiple weather data (e.g., temperature, humidity, wind speed, direction) at hourly intervals. We use $Q=\{{q}_{i}{\}}_{i=1}^{n}$ to denote the monitoring readings data from all stations.

### POI data

Besides, each monitoring station ${s}_{i}$ contains rich information on geographical features. We collect data on different types of points of interest (e.g., factories, residential areas, commercial areas) in the area around each monitoring station, and $P=\{{p}_{i}{\}}_{i=1}^{n}$ denotes the set of POIs for all stations.

### Time information

Each timestamp contains rich temporal information, which can respond to people's travel situation time information and thus assist in air quality prediction. We collected three kinds of time information: hour/day, day/week, and month. ${s}_{i}\in W$ denotes the set of time information of all monitoring stations.

### Problem definition

Given historical time data with a time window of length $T$, we take the historical monitoring readings $Q=({Q}^{t-T+1}, {Q}^{t-T+2}, \cdots ,{Q}^{t})$ for all stations as historical data, POI data $P$, and Time information $W$. The objective is to predict the value of some target pollutant at a future time step τ for all monitoring stations $S$ within the city, denoted by $\widehat{y}=({\widehat{y}}^{T+1}, {\widehat{y}}^{T+2},\cdots ,{\widehat{y}}^{T+\tau })$.

$$f(Q;P;W;\theta )\to \widehat{y}$$

where $\theta$ denotes all the parameters to be learned in the mapping function $f(\cdot )$.

## Proposed method

**Figure 2:**
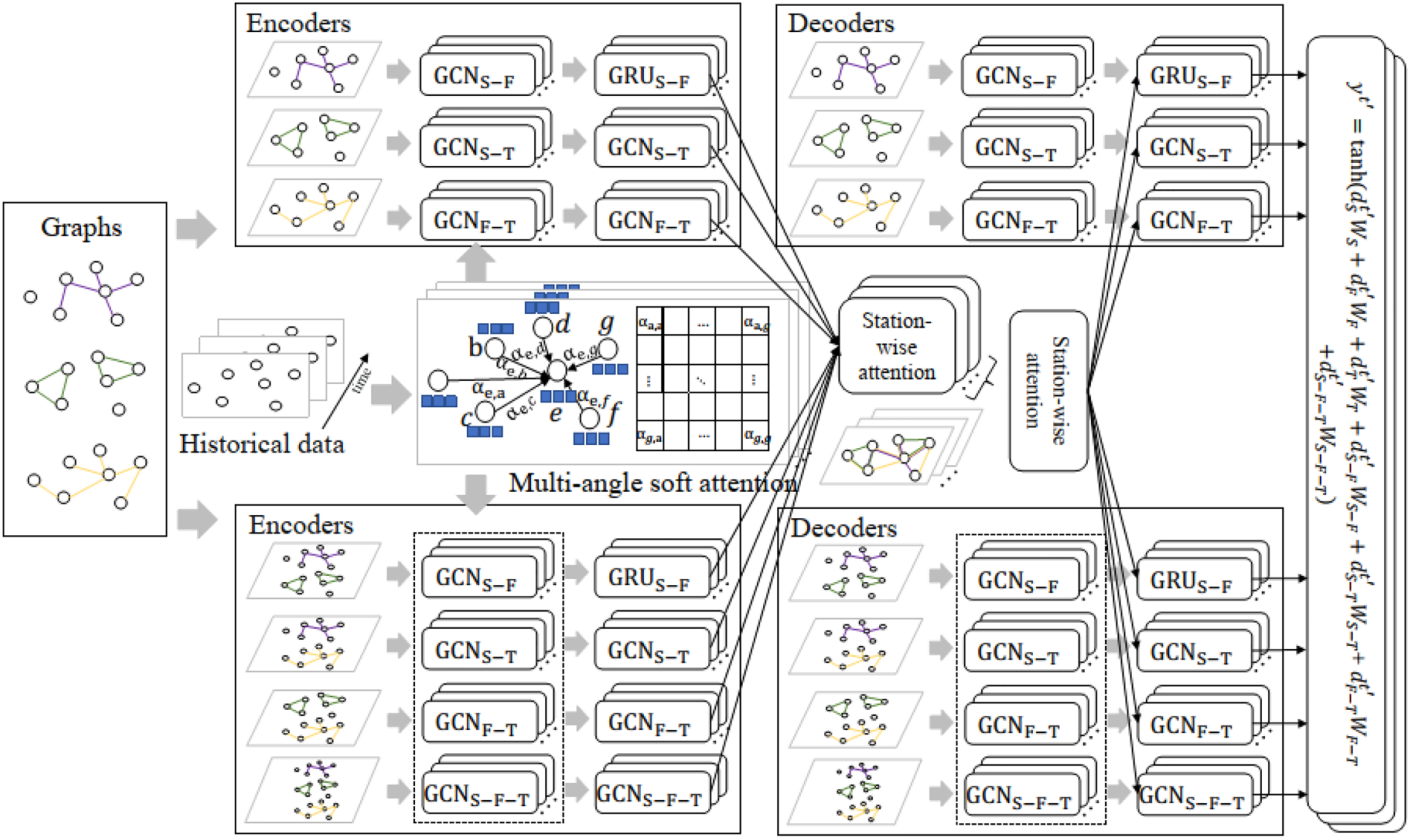
The overview framework of STAA-GCN.

This section describes in detail the modules of each part of our proposed STAA-GCN, and the overview framework of STAA-GCN is shown in Fig. 2.

### Graph generation

This section describes the graph generation of three specific types of inter-station relationships. We no longer generate new relationship graphs for the shared inter-station relationships, which rely on the three specific graphs to extract spatial dependencies. Formally, we generate graphs ${G}_{S}=\{V,{E}_{S},{A}_{S}\}$ based on spatial proximity spatial between stations, graphs ${G}_{P}=\{V,{E}_{P},{A}_{P}\}$ based on functional similarity, and graphs ${G}_{T}=\{V,{E}_{T},{A}_{T}\}$ based on temporal pattern similarity^11^.

#### Spatial proximity graph

In general, the closer two things are to each other, the stronger the correlation. We generate a spatial proximity graph using the physical spatial distance between two stations to achieve edge connectivity and assign weights.

$${A}_{{s}_{ij}}=\left\{\begin{array}{l}dist\left({v}_{i}, {v}_{j}\right)<{\alpha }_{S}\\ 0, otherwise\end{array}\right.$$

where the threshold realizes the connectivity between stations $i$ and $j$, the edge weights are calculated using the inverse of the physical space distance.

#### Functional similarity graph

Intuitively, when the POI around the two stations is relatively similar, there is a strong correlation between their air quality. When two stations are surrounded by industrial parks, they must have high air quality readings. And they may be strongly correlated on certain pollutants, such as industrial parks with similar production. We consider the similarity between stations with similar functions, then generate a functional similarity graph.

$${A}_{{P}_{ij}}=\left\{\begin{array}{l}sim\left({p}_{i}, {p}_{j}\right)>{\alpha }_{F}\\ 0, otherwise\end{array}\right.$$

where the threshold realizes whether the connectivity between stations $i$ and $j$, the edge weights are calculated using the Euclidean Distance between the POI vectors of stations $i$ and $j$.

#### Temporal pattern similarity graph

In addition to the two inter-station relationships mentioned above, there are potential temporal correlations between stations. We use the monthly average as a criterion for correlation:

$${A}_{{T}_{ij}}=\left\{\begin{array}{l}sim\left({t}_{i}, {t}_{j}\right)>{\alpha }_{T}\\ 0, otherwise\end{array}\right.$$

similarly, the edge weights are calculated using the Pearson Correlation Coefficient between the temporal patterns of stations $i$ and $j$.

### Complete spatiotemporal relationship interaction modules

We focus on complete spatiotemporal dependencies interaction modeling. Interactions between stations can be associated with one or more spatiotemporal dependencies. Using seven sets of encoders, we first capture the seven spatial relationships' spatiotemporal dependencies. Then, we employ a station-level attention mechanism to learn the interaction process between monitoring stations. In addition, air pollution's easy diffusion and dynamics cannot be ignored, and we design multi-angle soft attention to capture the pairwise dependencies between stations.

#### Multi-angle soft attention

First, air pollution is an aerosol mixture that exhibits a dynamic and easily diffusible nature in space, and it is inappropriate to ignore the unequal dynamic dependencies between stations. Second, the dependencies between stations are complex and diverse, and it is unconvincing to consider them simply from the intrinsic station data. To this end, we propose to capture the dynamic influence relationships between stations from multi-angle adaptively. Specifically, we first project station inherent data into multiple semantic spaces for enriching their semantic representations. Second, the influence of stations may vary nonlinearly. We nonlinearly map different semantic space information and obtain the final influence weights by computing them as the mean of the influence weights of all semantic spaces between stations.

For all stations $S$ at each time step $t$, we first concatenate its monitoring data (e.g., air pollutants and weather data), POI data, and time information as inputs ${X}^{t}\in {R}^{n\times d}$, the weights of multi-angle soft attention are:

$$({{x}_{i}^{m,t})}{\prime}={V}_{i}^{m}\cdot \sigma ({W}_{i}^{m}\cdot {x}_{i}^{t}+{b}_{i}^{m})$$

$${\alpha }_{i,j}^{t}=\frac{1}{M}\sum_{1}^{M}softmax(({{x}_{i}^{m,t})}{\prime})=\frac{1}{M}\sum_{1}^{M}\frac{{V}_{i}^{m}\cdot \sigma ({W}_{i}^{m}\cdot {x}_{i}^{t}+{b}_{i}^{m})}{\sum_{j=1}^{N}{V}_{j}^{m}\cdot \sigma ({W}_{j}^{m}\cdot {x}_{j}^{t}+{b}_{j}^{m})}$$

where ${W}_{i}^{m}\in {R}^{d\times {d}{\prime}}$, ${V}_{i}^{m}\in {R}^{{d}{\prime}\times 1}$, ${b}_{i}^{m}\in {R}^{d}$ are trainable parameters, $\sigma$ is the $tanh$ activation function, and $m\in M$ denotes the ${m}_{th}$ hidden space of the projection. Here, ${x}_{i}^{t}\in {R}^{d}$, ${x}_{j}^{t}\in {R}^{d}$ represent the observations at station $i,j$ at the ${t}_{th}$ moment. We recombine the computed weights among all stations into a weight matrix with ${E}^{t}\in {R}^{N\times N}$.

#### Specific graph convolutional unit

We capture specific types of inter-station relationships with their graphs, which ${r}_{spe}\epsilon \{S, F,T\}$ denotes any specific types of inter-station. Specifically, inspired by the success of Li and Kipf^5,7,23^ on graph convolution, combined with the weight matrix E, we use graph convolution in the vertex domain to aggregate K-hop neighbor information.

Given the observation ${X}^{t}\in {R}^{N\times d}$ at step $t$, we perform the Hadamard product operation on all stations weight matrices ${E}^{t}\in {R}^{N\times N}$ learned in Multi-angle soft attention with the weighted symmetric adjacency matrix ${A}_{sp}\in {R}^{N\times N}$. Finally, we aggregate the k-hop neighbor information as follows:

$$\tilde{X}_{{r_{{spe}} }}^{t}  = \mathop \sum \limits_{{k = 1}}^{K} \left( {\left( {D_{{r_{{spe}} }}^{t} } \right)^{{ - 1}} A_{{r_{{spe}} }}  \odot E^{t} } \right)^{k} X^{t} W_{{r_{{spe}} }}^{k}$$

where ${W}_{{r}_{spe}}^{k}\in {R}^{d\times d}$ is the trainable parameter, $k$ denotes k-hop neighbor reachable, and $\odot$ is the Hadamard product. ${\left({D}_{{r}_{spe}}^{t}\right)}^{-1}$ is the diagonal matrix of $A_{{r_{spe} }} \odot E^{t}$.

#### Shared graph convolutional unit

Intuitively, there are shared inter-station relationships in air quality prediction. We design a Bi-level sharing strategy to extract their relational features effectively. In this paper, shared inter-station relationships are divided into two categories, i.e., the combination of two arbitrary specific types of shared inter-station relationships and the combination of three specific types of shared inter-station relationships.

#### Shared graph convolution kernel strategy

**Figure 3:**
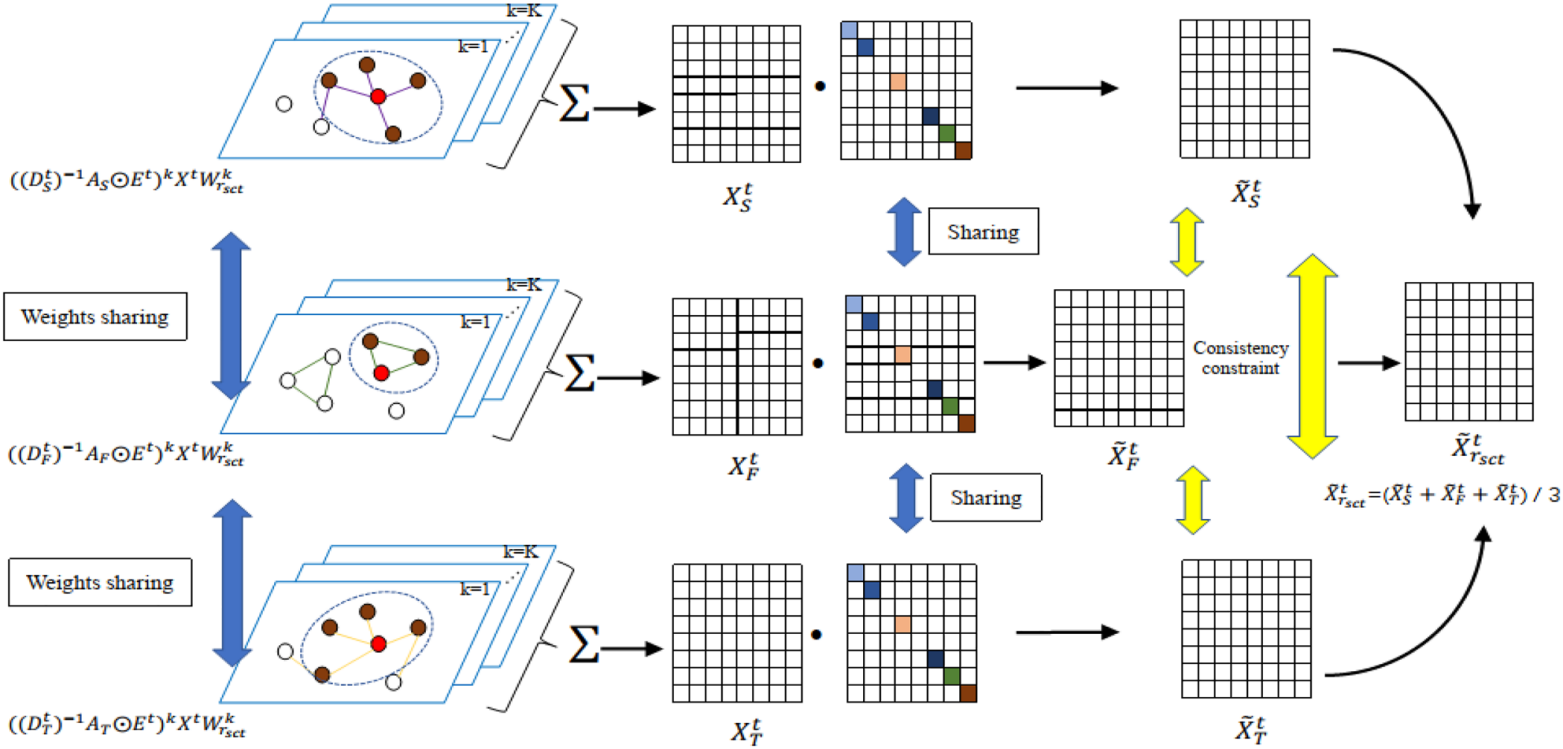
Diagram of Bi-level sharing strategy for three specific types of shared inter-station relationships.

We first train a set of shared graph convolution kernels to extract the features of shared inter-station among different spaces. For the combination of three, which ${r}_{sct}\epsilon \{S-F-T\}$, the spatial proximity graph ${G}_{S}$, the functional similarity graph ${G}_{P}$, and the temporal pattern similarity graph ${G}_{T}$ are taken as input; For the combination of the combination of two arbitrary, which ${r}_{sca}\epsilon \left\{S-F, S-T, F-T\right\}$, the graph used is consistent with the relationship of any combination. Here we take an example as the combination of three, and the overall process is shown in Fig. 3. The shared graph convolution kernel strategy is formalized as follows:

$$\left\{ {\begin{array}{*{20}c}    {X_{S}^{t}  = \mathop \sum \limits_{{k = 1}}^{K} \left( {\left( {D_{S}^{t} } \right)^{{ - 1}} A_{S}  \odot E^{t} } \right)^{k} X^{t} W_{{r_{{sct}} }}^{k} }  \\    {X_{F}^{t}  = \mathop \sum \limits_{{k = 1}}^{K} \left( {\left( {D_{F}^{t} } \right)^{{ - 1}} A_{F}  \odot E^{t} } \right)^{k} X^{t} W_{{r_{{sct}} }}^{k} }  \\    {X_{T}^{t}  = \mathop \sum \limits_{{k = 1}}^{K} \left( {\left( {D_{T}^{t} } \right)^{{ - 1}} A_{T}  \odot E^{t} } \right)^{k} X^{t} W_{{r_{{sct}} }}^{k} }  \\   \end{array} } \right.$$

where ${W}_{{r}_{sct}}^{k}\in {R}^{d\times d}$ are trainable parameters. ${X}_{S}^{t},{X}_{F}^{t},{X}_{T}^{t}$ are the station features extracted from ${G}_{S}$, ${G}_{P}$, and ${G}_{T}$ using shared graph convolution.

#### Shared diagonal matrix strategy

Not all stations have combinatorial shared dependencies with each other, and we use a set of shared diagonal matrices to remove irrelevant station feature information. The intuition behind the shared diagonal matrix is that using the shared graph convolution kernel, the feature information with shared dependencies dominates. We remove the sites with common weaker representation information by a set of shared diagonal matrices.

Specifically, diagonal matrix sharing is a set of shared sparse diagonal weight matrices. We first apply ${L}_{1}$ regularization to the diagonal weight matrix to remove irrelevant station feature information, so the sum of diagonal weight values is relatively small. Then by setting a threshold, we reset the station feature representation with consistently smaller weights in the three sets of feature representations to zero as follows:

$$\left\{\begin{array}{c}{\widetilde{X}}_{S}^{t}={X}_{S}^{t}{W}_{D}^{t}\\ {\widetilde{X}}_{F}^{t}={X}_{F}^{t}{W}_{D}^{t}\\ {\widetilde{X}}_{T}^{t}={X}_{T}^{t}{W}_{D}^{t}\end{array}\right.$$

Finally, we obtain the shared relation inter-station dependency feature representation as follows:

$${\widetilde{X}}_{{r}_{sct}}^{t}=({\widetilde{X}}_{S}^{t}+{\widetilde{X}}_{F}^{t}+{\widetilde{X}}_{T}^{t})/3$$

We adopt the Bi-level sharing strategy similar to that used for the three specific types of shared inter-station relationships for extracting the relationship between two arbitrary combinatorial stations. The difference is that two arbitrary are about sharing two arbitrary graphs, not three graphs, such as the spatial proximity graph and the functional similarity graph.

#### Temporal autocorrelation unit

We use the Gated Recurrent Units (GRU) on the temporal dependencies to capture the correlations on the temporal domain of each spatial dependence. Given any station spatial dependencies after learning at the current time step $t$ denoted ${\widetilde{X}}_{r}^{t}\epsilon \{{{r}_{spe}\cup r}_{sct}\cup {r}_{sca}\}$, combined with the hidden state ${h}^{t-1}$ at the previous step $t-1$, we compute the hidden state $h$ at time step $t$ as follows:

$$\left\{\begin{array}{c}{h}^{t}=\left(1-{z}^{t}\right)\odot {h}^{t-1}+{z}^{t}\odot {\widetilde{h}}^{t}\\ {r}^{t}=\sigma ({W}^{r}[{h}^{t-1}||{\widetilde{X}}_{r}^{t}] +{b}^{r})\\ {z}^{t}=\sigma ({W}^{z}[{h}^{t-1}||{\widetilde{X}}_{r}^{t}] +{b}^{z})\\ {\widetilde{h}}^{t}=tanh({W}^{\widetilde{h}}[{r}^{t}\odot {h}^{t-1}||{\widetilde{X}}_{r}^{t}] +{b}^{\widetilde{h}})\end{array}\right.$$

where ${z}^{t}$, ${r}^{t}$ denote the update gate and reset gate for controlling the inflow of previous information and forgetting the previous history information, respectively. Trainable parameters. ${W}^{r}\in {R}^{(d+h)\times d}$, ${W}^{z}\in {R}^{(d+h)\times d}$, ${W}^{\widetilde{h}}\in {R}^{(d+h)\times d}$, ${b}^{r}\in {R}^{d}$, ${b}^{z}\in {R}^{d}$, ${b}^{\widetilde{h}}\in {R}^{d}$ are trainable parameters, respectively.

#### Station-level attention fusion

Now we have seven spatiotemporal dependencies hidden states $M\{{\widetilde{h}}_{S}^{t}, {\widetilde{h}}_{F}^{t},{\widetilde{h}}_{T}^{t},{\widetilde{h}}_{S-F}^{t},{\widetilde{h}}_{S-T}^{t},{\widetilde{h}}_{F-T}^{t},{\widetilde{h}}_{S-F-T}^{t},\}$. We considered that the spatiotemporal dependencies between two stations could be one or more related spatiotemporal relationships. For this reason, we designed station-level attention fusion to learn the interaction process between stations:

$${h}_{m}^{i,t}={V}_{m}^{t}\mathrm{tanh}({W}_{m}^{t}\cdot {\left({\widetilde{h}}_{m}^{i,t}\right)}^{T}+{b}_{m}^{t})$$

$${\alpha }_{m}^{i, t}=softmax\left({h}_{m}^{i,t}\right)=\frac{\mathrm{exp}({h}_{m}^{i,t})}{\sum_{m\in M}\mathrm{exp}({h}_{m}^{i,t})}$$

$${h}^{i,t}=\sum_{m\in M}{\alpha }_{m}^{i, t}\cdot {h}_{m}^{i,t}$$

where ${W}_{m}^{t}\in {R}^{d\times {d}{\prime}}$, ${V}_{m}^{t}\in {R}^{{d}{\prime}\times 1}$, ${b}_{m}^{t}\in {R}^{d}$ are trainable parameters.

### Spatiotemporal feature extraction module

The correlation between the target series and its associated historical series is dynamic^10^. Therefore, before capturing the spatiotemporal dependence, we use temporal-level attention to capture the correlation between ${d}_{m}^{{t}{\prime}}$ and $\{{h}^{1},\cdots ,{h}^{T}\}$ in an adaptive manner and capturing the multiple spatiotemporal dependence from the complete spatiotemporal relationship, which ${d}_{m}^{t}\in \{{d}_{s}^{{t}{\prime}}, {d}_{F}^{{t}{\prime}}, {d}_{T}^{{t}{\prime}}, {d}_{S-F}^{{t}{\prime}}, {d}_{S-T}^{{t}{\prime}}, {d}_{F-T}^{{t}{\prime}},{d}_{S-F-T}^{{t}{\prime}}\}$. The ${d}_{m}^{{t}{\prime}}$ is decoded at each future time step ${t}{\prime}$ with similar to encoders.

$${\widetilde{d}}_{m}^{{t}{\prime}}={V}_{m}\mathrm{tanh}({W}_{m}\cdot {\left({d}_{m}^{{t}{\prime}}\right)}^{T}+{b}_{m})$$

$${\alpha }_{m}^{t}=softmax\left({\widetilde{d}}_{m}^{{t}{\prime}}\right)=\frac{\mathrm{exp}({\widetilde{d}}_{m}^{{t}{\prime}})}{\sum_{t\in T}\mathrm{exp}({h}^{t})}$$

$${c}_{m}^{t}=\sum_{T}{\alpha }_{m}^{t}\cdot {h}^{t}$$

where ${W}_{m}\in {R}^{d\times {d}{\prime}}$, ${V}_{m}\in {R}^{{d}{\prime}\times 1}$, ${b}_{m}\in {R}^{d}$ are trainable parameters. ${c}_{m}^{t}$ is contextual feature used as input to ${d}_{m}^{{t}{\prime}}$ and then update ${d}_{m}^{{t}{\prime}}$.

For the multi-step predictions, we aggregate multiple spatiotemporal dependencies ${d}_{m}^{{t}{\prime}}$ with a gating mechanism.

$${\widehat{y}}^{{t}{\prime}}=\mathrm{tanh}({d}_{s}^{{t}{\prime}}{W}_{S}+{d}_{F}^{{t}{\prime}}{W}_{F}+{d}_{T}^{{t}{\prime}}{W}_{T}+{d}_{S-F}^{{t}{\prime}}{W}_{S-F}+{d}_{S-T}^{{t}{\prime}}{W}_{S-T}+{d}_{F-T}^{{t}{\prime}}{W}_{F-T}+{d}_{s-F-T}^{{t}{\prime}}{W}_{S-F-T} )$$

where ${W}_{S}\in {R}^{d}$, ${W}_{F}\in {R}^{d}$, ${W}_{T}\in {R}^{d}, {W}_{S-F}\in {R}^{d}$, ${W}_{S-T}\in {R}^{d}$, ${W}_{F-T}\in {R}^{d}, {W}_{S-F-T}\in {R}^{d}$ are trainable parameters.

### Objective function

#### Regularization for multiple inter-station relationships

With the seven inter-station relationships modeling, which will lead to large number of parameters in the graph. To ensure the trainability of the model while reducing the risk of overfitting, we introduce basis decomposition:

$${W}_{r}^{l}=\sum_{b=1}^{B}{a}_{rb}^{l}{V}_{b}^{l}$$

where $r\epsilon \{{{r}_{spe}\cup r}_{sct}\cup {r}_{sca}\}$, ${V}_{b}^{l}$ is the share basis matrix between different convolution kernels, ${a}_{rb}^{l}$ is the linear coefficients of ${V}_{b}^{l}.$

#### Regularization for sparse of shared diagonal matrix

To get closer to the set threshold more efficiently, we apply ${L}_{1}$ regularization to the sum of diagonal weight values close to zero.

$${\mathcal{L}}_{{S}_{S-F}}=\sum_{w\in {W}_{{S}_{S-F}}}|w|$$

$${\mathcal{L}}_{{S}_{S-T}}=\sum_{w\in {W}_{{S}_{S-F}}}|w|$$

$${\mathcal{L}}_{{S}_{F-T}}=\sum_{w\in {W}_{{S}_{S-F}}}|w|$$

$${\mathcal{L}}_{{S}_{S-F-T}}=\sum_{w\in {W}_{{S}_{S-F}}}|w|$$

#### Consistency constraint

For shared inter-station relationships, we obtain the shared feature with ${L}_{2}$ regularization to consistency constraint between features. As following:

$${\mathcal{L}}_{{C}_{S-F}}={|{\widetilde{X}}_{S}^{t}-{\widetilde{X}}_{F}^{t}|}^{2}$$

$${\mathcal{L}}_{{C}_{S-T}}={|{\widetilde{X}}_{S}^{t}-{\widetilde{X}}_{T}^{t}|}^{2}$$

$${\mathcal{L}}_{{C}_{F-T}}={|{\widetilde{X}}_{F}^{t}-{\widetilde{X}}_{T}^{t}|}^{2}$$

$${\mathcal{L}}_{{C}_{S-F-T}}={|{\widetilde{X}}_{S}^{t}-{\widetilde{X}}_{F}^{t}|}^{2}+{|{\widetilde{X}}_{S}^{t}-{\widetilde{X}}_{T}^{t}|}^{2}+ {|{\widetilde{X}}_{F}^{t}-{\widetilde{X}}_{T}^{t}|}^{2}$$

Essentially, air quality prediction is a matter of regression. Therefore we use the root mean square error (MSE) as a loss function between the predicted and ground truth.

$${\mathcal{L}}_{P}=\sum_{t=1}^{\tau }{||{y}^{T+t}-{y}^{T+t}||}_{2}^{2}$$

The total loss of the proposed architecture as following:

$$\mathcal{L}= {\mathcal{L}}_{P}+\gamma \left({\mathcal{L}}_{{ES}_{S-F}}+{\mathcal{L}}_{{DS}_{S-F}}\right)+\delta \left({\mathcal{L}}_{{ES}_{S-T}}+{\mathcal{L}}_{{DS}_{S-T}}\right)+\varepsilon \left({\mathcal{L}}_{{ES}_{F-T}}+{\mathcal{L}}_{D{S}_{F-T}}\right)+\epsilon \left({\mathcal{L}}_{{ES}_{S-F-T}}+{\mathcal{L}}_{{D}_{S-F-T}}\right)+ \zeta \left({\mathcal{L}}_{{EC}_{S-F}}+{\mathcal{L}}_{D{C}_{S-F}}\right)+\eta \left({\mathcal{L}}_{{EC}_{S-T}}+{\mathcal{L}}_{D{C}_{S-T}}\right)+\vartheta \left({\mathcal{L}}_{E{C}_{F-T}}+{\mathcal{L}}_{D{C}_{F-T}}\right)+ \iota ({\mathcal{L}}_{E{C}_{S-F-T}}+{\mathcal{L}}_{{DC}_{S-F-T}})$$

where $\gamma$,$\delta ,\varepsilon , \epsilon , \zeta ,\eta ,\vartheta$, and $\iota$ are the hyper-parameters. Since the decoders have similar modules with encoders, a double loss is generated in the sparse of shared diagonal matrix and the consistency constraint, where ${\mathcal{L}}_{E}$, ${\mathcal{L}}_{D}$ denote the losses of encoders, decoders, respectively.

## Data availability

### Air quality dataset

We conducted experiments on three real-world air quality datasets. Beijing air quality dataset is available in Chinses Air Quality Historical Data, https://quotsoft.net/air/. Tianjin air quality dataset is available in Urban Computing, http://urban-computing.com/data/Data-1.zip. And London air quality dataset is available in the Artificial Intelligence Competition Learning Platform, https://www.biendata.net/competition/kdd_2018/. The datasets widely used in the air quality prediction literature. The Beijing air quality dataset is from 01/2016 to 01/2018, including PM2.5, PM10, SO_2_, NO_2_, and O_3_; The Tianjin air quality dataset is from 01/2014 to 04/2015, including PM2.5, PM10, SO2, NO2, O3, and CO; The London air quality dataset is from 01/2017 to 03/2018, including PM2.5, PM10, and NO_2_.

### Meteorology and POI data

Historical meteorology data and weather forecasts are used to improve the accuracy of predictions^6,11^. We selected five attributes of grid weather datasets, including temperature, humidity, wind speed, wind-u, and wind-v. The gridded weather dataset for Beijing is available in the Global Data Assimilation System, https://www.ncdc.noaa.gov. The gridded weather dataset for Tianjin is available in Urban Computing, http://urban-computing.com/data/Data-1.zip. The gridded weather dataset for London is available in the Artificial Intelligence Competition Learning Platform, https://www.biendata.net/competition/kdd_2018/. Following the ATGCN^11^, POI data for all three datasets are available in Amap, https://lbs.amap.com/api/webservice/download. POI data including 12 categories from Beijing, 20 from Tianjin and 8 from London. In addition, Following previous work^6^, we also used temporal information features to support air quality prediction, including hours/day, days/week, and months. The statistics of the datasets are shown in Table 2.

**Table 2:** Statistics of the datasets used in this paper.

Description	Beijing	Tianjin	London
# Of air quality stations	35	26	13
# Of air quality timespan	01/2016–01/2018	01/2014–04/2015	01/2017–03/2018
# Of air quality features	5	6	3
# Of air quality records	1,079,040	214,760	141,661
# Of POI categories	12	20	8
# Of meteorology features	4	5	5
# Of time features	3	3	3

## Experiments

### Implementation details

We use linear interpolation extensively for data pre-processing. To ensure the authenticity and reliability of the experiments, we used a non-overlapping 8:1:1 ratio for the training, validation, and test sets.

STAA-GCN and all the deep learning model experiments were run on Python 3.7.4, PyTorch 1.9.1 environment. For STAA-GCN, the cell size of GRU and the hidden states size in the graph convolution layer to 64. We aggregate 2-hop neighbor information with K = 2.

The learning parameters used initialize with uniform distribution. Our model is trained by objective function with the Adam optimizer, and the learning rate is set to 0.001. We set the batch size to 256 for Beijing, Tianjin and London datasets. We prevent overfitting the training data with the early stop mechanism in the training phase, in which patience is 15. The time window T we set to 12 and the prediction step τ to 6. We use PM2.5 as the prediction target because it is always the most important of all pollutants. Our code is publicly available at https://github.com/349117955/STAAGCN.

### Evaluation metrics and baselines

We use Root Mean Squared Error (RMSE), Mean Absolute Error (MAE), and Mean Absolute Percentage Error (MAPE), widely used for air quality prediction tasks, to measure model performance. The model's validity is verified by comparing it with the following seven methods.

**HA** The average of the historical time steps was used as the predicted value of PM2.5.

**SVR** Learned linear relationships between historical time series to perform multi-step forecasting.

**Seq2seq** Encoder-Decoder based methods have been widely used in multi-step forecasting.

**MGED-Net**^6^ Air quality prediction model for multi-feature relationship learning using multi-group feature fusion approach.

**Graph WaveNet**^24^ In spatiotemporal prediction, GCN models spatial dependencies with spatiotemporal data.

**ATGCN**^11^ Multi-graph based air quality prediction methods, using multiple sets of Encoder-Decoder to model the Inter-station relationships.

### Experimental results

#### Performance comparison

Table 3 shows the experimental prediction results of all methods and the effect of different inter-station relationships, which measure the method's performance using the MAE and RMSE over the next 6 h. We observe that STAA-GCN achieves state-of-the-art experimental results in all methods using two evaluation metrics. All deep learning methods outperform the traditional methods, which demonstrate the non-linear between sequences. The experimental results of Seq2seq, Graph WaveNet, and ATGCN methods illustrate the effectiveness of the spatial modeling approach. STAA-GCN outperforms all the methods because the interaction between stations is fully considered, and spatiotemporal features are effectively extracted.

**Table 3:** The prediction performance of different methods and removal of different inter-station relationships are compared on three datasets.

Model	Beijing			Tianjin			London		
	MAE	RMSE	MAPE	MAE	RMSE	MAPE	MAE	RMSE	MAPE
HA	20.68	32.10	0.89	34.44	49.66	0.83	5.37	7.76	0.84
SVR	22.35	27.80	0.83	28.45	38.98	0.84	7.94	9.15	0.91
Seq2seq	14.90	22.51	0.62	19.41	30.21	0.43	5.32	7.22	0.78
MGED-Net	14.85	22.12	0.68	18.42	28.41	0.45	5.15	7.11	0.69
Graph WaveNet	14.75	22.24	0.59	16.81	26.49	0.40	4.16	5.80	0.55
ATGCN	14.73	22.05	0.58	16.91	26.21	0.39	4.44	6.31	0.58
-w/o S	14.15	21.89	0.51	16.31	25.51	0.39	4.21	5.83	0.56
-w/o F	14.27	21.95	0.57	16.65	25.79	0.39	4.13	5.76	0.53
-w/o T	14.18	21.92	0.52	16.71	25.87	0.40	4.14	5.77	0.54
-w/o S-F	14.51	21.93	0.53	16.25	25.42	0.38	4.12	5.79	0.55
-w/o S-T	14.53	21.94	0.53	16.52	25.65	0.39	4.09	5.79	0.50
-w/o F-T	14.28	21.91	0.52	16.69	25.91	0.42	4.11	5.76	0.49
-w/o S-F-T	14.54	21.98	0.55	16.72	25.97	0.41	4.13	5.79	0.51
STAA-GCN	**13.97**	**21.51**	**0.49**	**16.13**	**25.37**	**0.37**	**4.07**	**5.73**	**0.48**

To verify that multiple inter-station relationships exist, we conduct experiments on multiple sets of variants of STAA-GCN on three datasets. We remove different types of inter-station relationships, including spatial proximity (S), functional similarity (F), temporal pattern similarity (T), spatial adjacency-functional similarity (S-F), spatial adjacency-temporal pattern similarity (S-T), functional similarity-temporal pattern similarity (F-T), and spatial adjacency-functional similarity-temporal pattern similarity (S-F-T). We observed that the removal of each inter-station relationship causes a degradation of the model performance, which suggests the validity of these inter-station relationships.

#### Ablation experiment

To study the effectiveness of each component in our model, we conduct experiments on four variants of STAA-GCN on three datasets. (1) STAA-GCN w/o STA-attn, which removes the station-level attention mechanism for building complete spatiotemporal interaction; (2) STAA-GCN w/o Mul-attn, which removes the multi-angle soft attention for capturing unequal weights between stations; (3) STAA-GCN w/o SA-matrix, which removes shared diagonal matrix for removing stations features without shared dependencies; (4) STAA-GCN w/o similar loss, which removes similarity constraints for extraction sharing representation. We tested the performance of different variants in agreement with the parameters of the STAA-GCN.

**Figure 4:**
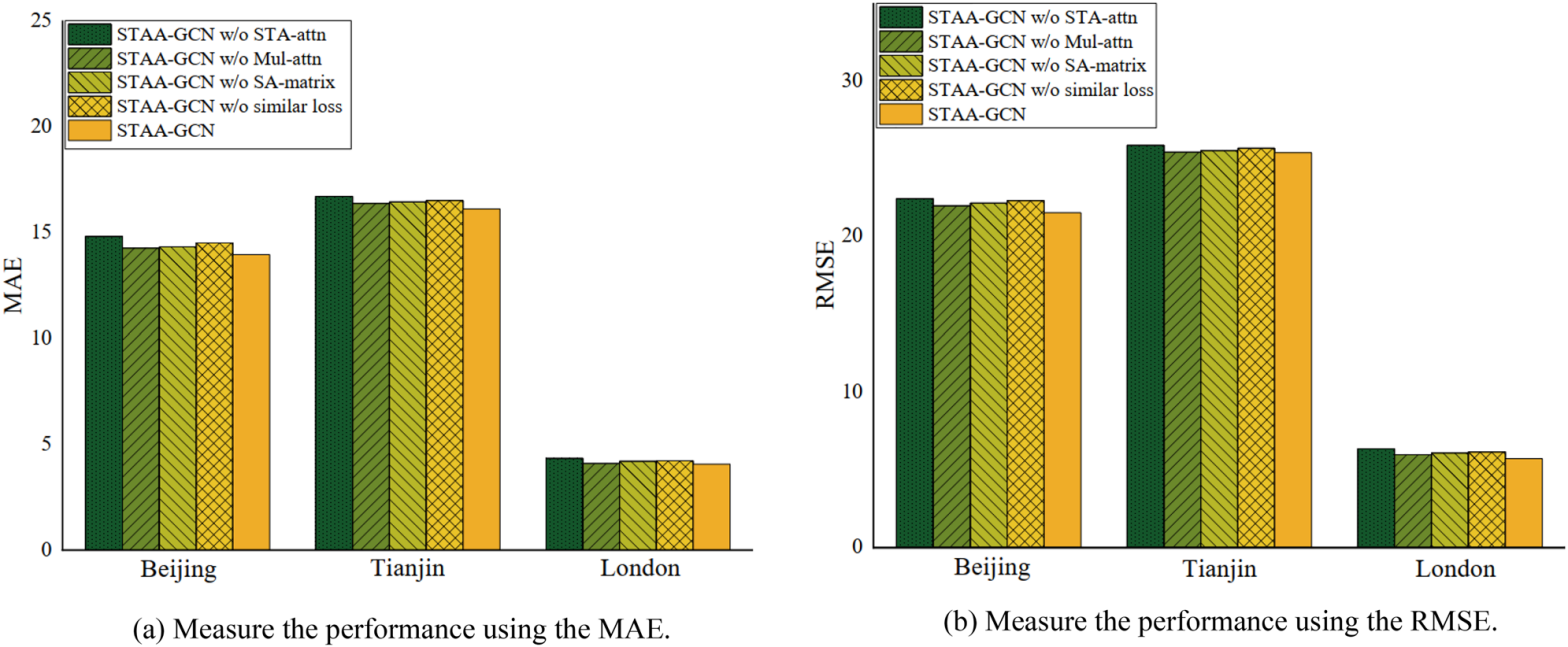
Ablation Experiment of STAA-GCN on three datasets.

Figure 4 shows the ablation Experiment of STAA-GCN with the method's performance using the MAE and RMSE on three datasets. First, the model performance declines sharply without complete spatiotemporal interaction. Second, variants without multi-angle soft focus obtained poorer results than STAA-GCN, which validates the validity of our approach and the existence of the unequal relationship between stations. For shared inter-station relations, we remove the shared diagonal matrix or similar sharing constraints, respectively, and the effectiveness of our designed scheme is illustrated with the experimental results.

#### Hyperparameter sensitivity

We conducted extensive experiments on three datasets to further study the sensitivity of parameters in our model.

**Figure 5:**
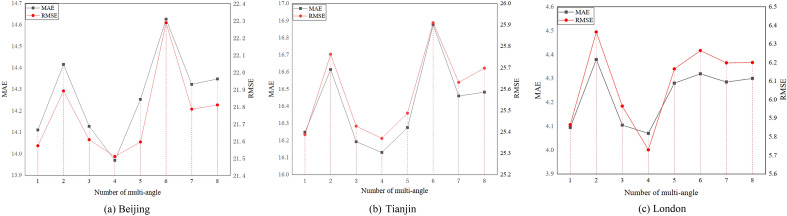
Effect of the number of multi-angle on three datasets.

First, we vary the number of multi-angle from 1 to 8 on three datasets. The results are shown in Fig. 5. As the number of multiple angles increases, the performance drops sharply, picks up at number three, levels off at number four, and then drops again. STAA-GCN obtains the best performance on three datasets with the number four. The cause of the above is that smaller quantities cannot capture multi-dimensional relationships, and more quantities introduce extraneous noise, leading to performance degradation.

**Figure 6:**
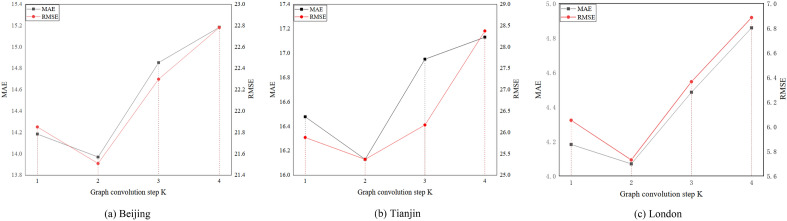
Effect of the graph convolution step K.

Then, we vary the number of the graph convolution step K from 1 to 4 on three datasets. The results are shown in Fig. 6. we can see that as the graph convolution step increases and then decreases. The best performance is step 2 on all datasets. The cause of the above is that a low or high graph convolution step can lead to fewer aggregated or irrelevant features aggregated from k-order neighbors.

**Figure 7:**
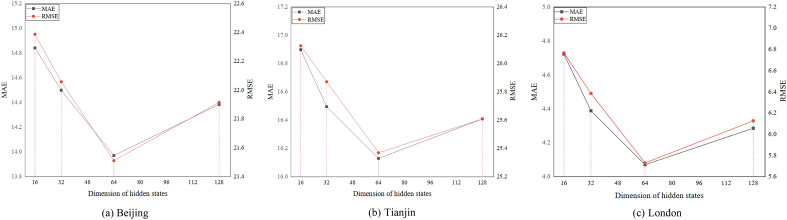
Effect of the dimension of hidden states.

After that, we vary the dimension of hidden states as {16, 32, 64, 128} on three datasets. The results are shown in Fig. 7. We can see that the best performance is 64. Generally, A hidden state that is too small will lose some information, while too large will cause poor performance due to overfitting.

**Figure 8:**
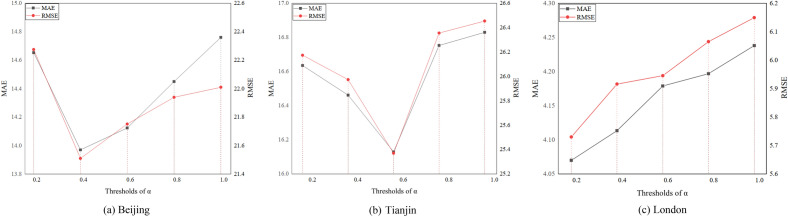
Effect of α.

Finally, we vary the threshold for removing irrelevant station feature information. We experiment by setting the thresholds to α for triple-shares, which α from 0.15 to 0.95. We test the validity of the parameters α, the results are shown in Fig. 8. we can see that the performance increases and then drops. The main reason is that shared dependencies exist only between certain stations.

## Conclusions and future work

This paper proposes a new deep learning model, the spatiotemporal adaptive attention graph convolution model STAA-GCN, for city-level air quality prediction. The core of STAA-GCN is its efficient extract multiple spatiotemporal features, which are extracted from the complete inter-station interactions between stations. Furthermore, this model also considers the inherent inequality relationship between stations. Our model achieves the best experimental results in a broad range of tests on all three publicly available datasets and demonstrates the importance of the complete spatiotemporal interactions for the extraction of spatiotemporal dependencies and the effectiveness of the seven inter-station relationships.

In the future, we would like to fully use more datasets from areas without air pollutant monitoring, such as fine-grained gridded weather datasets and POI datasets. The spread and dispersion of air pollution are unstable and mutable, related to the weather changes and environmental characteristics during transmission. Therefore, we will use the tensor decomposition as a basis, combined with a large amount of gridded influence factor data, to fill the areas where no monitoring data are available. That will further clarify the process of pollutant dispersion, rather than just passing information between monitoring stations, and we will achieve the improvement of the accuracy of existing models, thereby enabling a more accurate basis for people's healthy outdoor travel.
